# Practical Tracking Method based on Best Buddies Similarity

**DOI:** 10.34133/cbsystems.0050

**Published:** 2023-08-29

**Authors:** Haiyu He, Zhen Chen, Haikuo Liu, Xiangdong Liu, Youguang Guo, Jian Li

**Affiliations:** ^1^ School of Automation, Beijing Institute of Technology, Beijing, China.; ^2^ School of Mechatronical Engineering, Beijing Institute of Technology, Beijing, China.; ^3^ School of Electrical and Data Engineering, University of Technology Sydney, Sydney, Australia.

## Abstract

Visual tracking is a crucial skill for bionic robots to perceive the environment and control their movement. However, visual tracking is challenging when the target undergoes nonrigid deformation because of the perspective change from the camera mounted on the robot. In this paper, a real-time and scale-adaptive visual tracking method based on best buddies similarity (BBS) is presented, which is a state-of-the-art template matching method that can handle nonrigid deformation. The proposed method improves the original BBS in 4 aspects: (a) The caching scheme is optimized to reduce the computational overhead, (b) the effect of cluttered backgrounds on BBS is theoretically analyzed and a patch-based texture is introduced to enhance the robustness and accuracy, (c) the batch gradient descent algorithm is used to further speed up the method, and (d) a resample strategy is applied to enable the BBS to track the target in scale space. The proposed method on challenging real-world datasets is evaluated and its promising performance is demonstrated.

## Introduction

Visual tracking is a vital application in bionic robot technology [1–3], which endows robots with the ability to track a specified target by analyzing image data and to autonomously control their motion [4]. However, visual tracking poses unique challenges for robot vision, as the camera is usually fixed on the robot platform and the target may undergo nonrigid deformation because of the change of perspective caused by the relative motion between the robot and the target [5,6]. It results in dynamic blur and nonrigid deformation, which degrades the performance of the tracker. Moreover, because mobile robots often adopt embedded development environments [3], they cannot use graphics processing unit considering the demands of environmental adaptability and reliability. In this resource-constrained scenario, the limited computing resources have to compromise the computational efficiency of the tracker.

Visual tracking aims to continuously estimate the target states in the subsequent video frames given the initial bounding box of the target and the target image information. The visual tracking problem can also be viewed as a special case of the template matching problem. Therefore, various similarity measures for template matching have been applied to visual tracking to quantify the similarity between the template and the candidate image, such as normalized minimum mean square error [7,8], cosine similarity [9], and Bhattacharyya similarity [10].

Recently published best buddies similarity (BBS) [11] has achieved state-of-the-art performance in template matching problems. The BBS transforms the template and the target image into point sets and counts the number of best buddies pairs (BBP) among them. The BBS does not depend on the global information of the template and only finds the nearest neighbor of each point in the template point set, making it a robust solution for geometric deformation. Most researches on BBS focus on the template matching field [12–15]. However, such approaches do not pay much attention to the real-time visual tracking problem because of its high computational overhead. A practical tracker must strike a delicate balance between accuracy and real-time performance.

BBS uses the sliding window strategy for traversal searching. Although the original algorithm has proposed a caching scheme, there is still a lot of redundant computation. Reducing computation time to an acceptable level with a promising accuracy is an effective solution for its application in visual tracking. In this paper, to address this issue, a real-time and scale-adaptive tracking method is presented on the basis of BBS. The caching scheme proposed by the original work is also improved. Moreover, the reason why BBS is affected by cluttered backgrounds is theoretically demonstrated, and the patch-based texture is introduced to enhance the robustness. Meanwhile, the batch gradient descent (BGD) algorithm is employed to further accelerate the algorithm. Finally, the proposed method is successfully applied to scale space tracking by introducing a resample method. The main contributions of this paper are summarized as follows. A real-time and scale-adaptive best buddies tracker (RSBBT) is presented, which can handle scale space tracking by introducing a resample strategy, and achieves at least 10 times faster than the BBS when processing images with the same size. The proposed method is evaluated on challenging real-world datasets and shows its promising performance. A caching scheme based on hash-encoded patches is proposed, which does not depend on the sliding direction of the sliding window and can start from any direction and location. The processing speed of a single step also has about 10% improvement. The computation efficiency of BBS is optimized by adapting the improved caching scheme. The impact of cluttered background on BBS is theoretically demonstrated in this paper. Moreover, the patch-based texture features are introduced to address the problem of local multiple response peaks caused by cluttered backgrounds. Meanwhile, a naive BBS maximum response search method is proposed, which enhances the robustness and accuracy of BBS.

## Materials and Methods

### Related works

In recent years, many tracking methods have been proposed. Visual tracking methods can be roughly divided into 2 categories [16]: generative methods and discriminative methods.

The generative tracking algorithm does not consider the background information, constructs a model to represent the target, and then uses the model to search the target. Widely used generative methods include mean-shift [8], particle filter [17], optical flow [18], etc.

Discriminative methods consider the video target tracking problem as a supervised linear binary classification problem or a regression problem. The classifier is trained by extracting training samples from the target and background to detect objects within the search window. The representative discriminative algorithms are the support vector machines [19], deep neural networks [16], and correlation filters [7,20,21].

In its most basic form, online tracking boils down to template matching, where the goal is to find a given template in the current image. The target tracking method often depends on the definition of a similarity (distance) measurement, such as the mean square error in minimum output sum square error (MOSSE) [7], Bhattacharyya similarity in scale-adaptive mean-shift (ASMS) [22], etc.

BBS is a nonparametric robust similarity measurement method that counts the number of the nearest neighbor point pairs between 2 point sets in an unconstrained condition [11]. It can be shown that, for sufficiently large point sets, BBS converges to the chi-square distance. This method adopts the sliding window strategy to scan the whole image pixel by pixel with the same size as the template. Therefore, BBS has a high computational overhead. To reduce the redundant computation and manifest the principal features, a reuse scheme was proposed, including (a) distance computation reuse, (b) minimum operator load reduction, and (c) additional load reduction, which dramatically reduce the computation time. Although this caching scheme greatly reduces the computation time of BBS, it also restricts the BBS in a sliding window search framework, and thus, it is not suitable for visual tracking problem. As a result, the computation complexity is very high. To accelerate the algorithm, several algorithms have been employed to decrease the computation time.

Talmi et al. [23] introduced the concept of point set diversity. That is, the points in the template point set are uniquely determined, and every point in the template also has a uniquely determined nearest neighbor in the target point set. On the basis of this assumption, a similarity measurement function is designed to describe the diversity of the point set, and unilateral matching is used to reduce the computational complexity. It can be seen that the diversity of point set can improve the BBS method, but it did not give theoretical proof and cannot handle scale variation. To decrease the computation overhead of the sliding window strategy, a widespread adoption is the fast algorithm based on the winner-update strategy presented in [24]. The basic idea is to generate image pyramids that utilize an ascending lower bound list of the matching error to determine the temporary winner. However, BBS may fail when the template or search window is very small. Therefore, the image pyramid strategy may not be a good choice. Xia et al. [25] proposed the deformable best buddies similarity method based on BBS. Constraints such as multiscale combinatorial grouping; normalized cross correlation; and shape, size, and color appearance feature were introduced to discover potential target areas, and BBS was adopted to search for targets in the potential area to reduce computation. Oron et al. [26] embedded BBS into a particle filtering framework. A modification that lets BBS handle point sets of different size was proposed. The modified BBS can handle scale changes in the template size and can support a variable number of template images. The random sample strategy is adopted to reduce the computation. However, the particle filter framework, meanwhile, introduces a large amount of particle windows, which further increases computation complexity.

The BBS is a powerful similarity measure for visual tracking, as it can cope with complex geometric deformations and high levels of outliers. However, several challenges hinder the application of BBS for visual tracking, such as high computational overhead, limited scalability, and sensitivity to cluttered backgrounds. Therefore, a more efficient and robust visual tracking method based on BBS is developed in this paper.

## Best buddies similarity

The BBS is a nonparameter robust similarity method that can measure the similarity between a specific template and a candidate image. The number of nearest neighbors in the candidate point sets is counted implicitly according to the distance matrix. The BBS performs bidirectional matching without constraints and preliminary assumptions.

In the original work [11], a naive implementation was provided. That is, dividing the image into nonoverlapping patches with the size of *k* × *k* and then expanding the patches into column vectors with *d* dimensions. The RGB (red, green, blue) color feature is utilized to compute the distance matrix. Hence, the image patch is represented by a (*k* × *k*)*^d^* vector.

The bidirectional matching performed by BBS between 2 point sets *T*, *S* ∈ *ℝ^d^* requires going through all feature vectors. Hence, the distance matrix is defined as *D* = [*d_ij_*], where *i* and *j* represent the number of template and candidate vectors, respectively. With the given *D*, the minimal element in the *i*th row and the minimal element in the *j*th column represent the nearest neighbor of *p_i_* (i.e., *NN*(*t_i_*, *S*)) and the nearest neighbor of *s_i_* (i.e., *NN*(*s_i_*, *T*)), respectively. Then, the BBS is computed by counting the number of mutual nearest neighbors and dividing it by a constant. Formally,

$$\mathrm{bb}\left(s_{i},t_{i},S,T\right)=\left\{\begin{array}{cc} 1 & \mathrm{NN}\left(t_{i},S\right)=s_{i}\wedge \mathrm{NN}\left(s_{i},T\right)=t_{i},\\ 0 & \;\text{otherwise} \end{array}\right.$$ (1)

where *NN*(*t_i_*, *S*) = argmin_*s_i_* ⊂*S*_*d*(*t_i_*, *s_i_*) and *d*(*t_i_*, *s_i_*) are some distance measurements. The BBS between the point sets *S* and *T* is given by

$$B\left(x\right)=\frac{1}{N}\sum_{i=1}^{N}‍\sum_{j=1}^{N}‍\mathrm{bb}\left(s_{i},t_{i},S,T\right).$$ (2)

## Proposed algorithm

The original BBS computes the response values of all possible positions. It is essentially a traversal search in space. It can be seen that the problem of target tracking has its particularity. It is unnecessary to traverse the entire image. A searching algorithm such as the BGD method (or heuristic search algorithm) is employed to find the position with the maximum response value. Therefore, the exhaustive searching problem is converted to a mode-seeking problem.

### Patch-based caching scheme

The BBS is a similarity between 2 point sets, the distance between each point pair needs to be computed one by one, and there is a mount of redundant computation. Oron et al. [11] proposed a point set-based caching scheme to increase the memory in exchange for complexity, and the computational complexity is greatly reduced. The caching scheme needs to be improved to accelerate the search process and achieve a promising real-time performance. However, the caching scheme depends on the sliding direction of the window and should be computed pixel by pixel from top to bottom and left to right. This caching scheme is applied only to the sliding window strategy.

To address the mentioned issue, a patch-based caching scheme is proposed in this paper. For candidate image point set *S*, the *k* × *k* patch is used as the minimum computation unit, and the distance results are indexed by column. In practice, 2 hash tables *I* and *S* are maintained, which are defined as initial empty mappings from *d*-dimensional patches (corresponding to grid cells) to non-negative integers and *R^d^*, respectively. Among them, the index of each image patch is stored in *I*, and the distance results of all the template patches are stored in *S*.

**Fig. 1.**
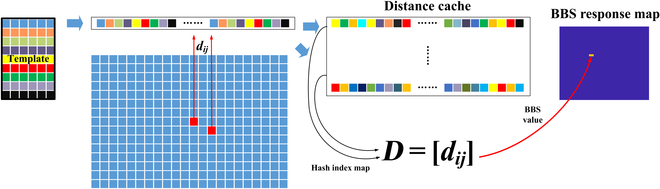
An overview of the caching scheme.

The patch-based caching scheme no longer depends on the sliding direction of the window and can be computed at any position in any direction. An overview of the proposed caching scheme is shown in Fig. 1.

### A naive implementation of RSBBT

In general, the template is given by a bounding box, which contains background pixels. The BBS is a statistical method, and hence, pixels belonging to the background would interfere with the BBS response map and lead to problems of drift and multipeak. The expectation BBS(*S*, *T*), over all possible samples of *S* and *T*, is the sum of the expectation BBP,

$$E\left(\mathrm{BBS}\left(S,T\right)\right)=\frac{1}{N}\sum_{i=1}^{N}‍\sum_{i=1}^{N}‍\mathrm{bb}\left(s_{i},t_{i},S,T\right)=N\cdot E_{\mathrm{BBP}}$$ (3)

It can be seen that *E*(BBS(*S*, *T*)) is linearly dependent on *E*_BBP_. Let *ε* represent the minimum distance in *D*, and *ε* > 0. When there exists at least one pair of points in *T*, let *d_ij_* < *ε*,*bb*(*s_i_*, *t_i_*, *S*, *T*) equals 1, that is,

$$E_{\mathrm{BBP}}=\iint ‍\mathrm{bb}\left(s_{i},t_{i}\right)\cdot \mathrm{pr}\left(\exists s_{i}\in S,d_{ij}<\varepsilon \right)\text{dSdT}$$ (4)

Assuming that there are *N* distributions in template point sets, and there are *K* different distributions among them, *K* < *N*, and pr(∃*t_i_* ∈ *T*, *d_ij_* < *ε*) denotes the probability that there exists point *t_i_* in *T* that is *ε*-close to *s_i_*. Formally, it is shown that, when *K* trends to *N*, pr(∃*t_i_* ∈ *T*, *d_ij_* < *ε*) decreases. pr(∃*t_i_* ∈ *T*, *d_ij_* < *ε*) is hard to describe in a numerically way. According to the total probability formula, pr(∃*t_i_* ∈ *T*, *d_ij_* < *ε*) is given by:

$$\mathrm{pr}\left(\exists t_{i}\in T,d_{ij}<\varepsilon \right)=1-\mathrm{pr}\left(\forall t_{i}\in T,d_{ij}\geq \varepsilon \right).$$ (5)

Hence, the probability that, for any point *t_i_* in *T*, *d_ij_* ≥ *ε* can be checked. pr(*t_i_* ∈ Φ_*s_i_*,*ε*_) is defined to denote the probability that *t_i_* belongs to the *ε*-hypersphere around *s_i_*,

$$\mathrm{pr}\left(t_{i}\in \Phi_{s_{i},\varepsilon }\right)=\iint ‍\ldots \int ‍f_{T}\left(t\right)\mathrm{dt},$$ (6)

where *f_T_*(*t*) denotes the cumulative distribution function of *T*. For any point sets, *f_T_*(*t*) is a multivariable distribution, which requires integration in all dimensions, which is very difficult. Fortunately, the actual integration value is not necessary, and the tendency deserves more attention, assuming that the distribution is continuous. The *f_T_*(*t*) is a smooth distribution function because *T* and *S* are finite and 0 < *ε*. Then,

$$0<\mathrm{pr}\left(t_{i}\in \Phi_{s_{i},\varepsilon }\right)<1$$ (7)

It can be found that *ε* is the minimum distance in *D*. Hence, at least 1 and, at most, *N − K* distributions fall into the *ε*-hypersphere of , then

$$\prod_{i=1}^{N-K}‍\left[1-\mathrm{pr}\left(t_{i}\in \Phi_{s_{i},\varepsilon }\right)\right]\leq \mathrm{pr}\left(\forall t_{i}\in T,d_{ij}\geq \varepsilon \right)\leq 1-\mathrm{pr}\left(t_{i}\in \Phi_{s_{i},\varepsilon }\right),$$ (8)

when $K\to N,\prod_{i=1}^{N-K}‍\left[1-\mathrm{pr}\left(t_{i}\in \Phi_{s_{i},\varepsilon }\right)\right]$ increases. Another issue can also be illustrated by the above proof, that is, similar point sets in the background will affect the response value of the BBS. Therefore, multiple response peaks may appear in the response map, making it difficult to find the target location.

**Fig. 2.**
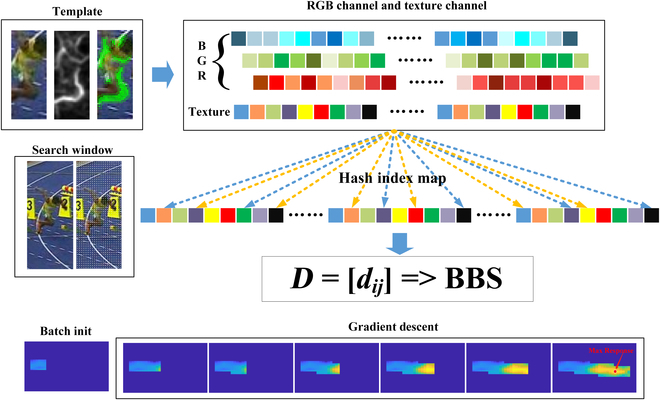
An overview of the naive implementation.

Oron et al. [26] have proved that when the size of the point sets does not match, the larger the size of *S*, the larger the BBS. That is, when *N* →  ∞ , pr(∃*s_i_* ∈ *S*, *d_ij_* < *ε*) tends to 1. Therefore, it is not a good strategy to adjust the size of the point set or modify the template (such as the template update strategy). Adjusting the size of the point set will lead to an increase or decrease of *N*. A simple modification may also cause an unknown change of *K*, thereby affecting the response of the BBS. To address the mentioned issues, the patch-based texture features are introduced. Meanwhile, a strategy to reduce the distribution type in the target template is adopted, thereby enhancing the robustness of the distribution in template point sets. An overview of the naive implementation is given in Fig. 2.

First, for the image patch of *k* × *k* × *d*, the gradient of the pixel at (*x*, *y*) is computed in the *d* dimensions, and the gradient value is used as a constraint to naive RGB color features,

$$|f_{d}\left(x,y\right)|=\text{mag}\left(\nabla f_{d}\left(x,y\right)\right),$$ (9)

$$|f_{d}\left(x,y\right)|=\sqrt{G_{x}{\left(x,y\right)}^{2}+G_{y}{\left(x,y\right)}^{2}},$$ (10)

*G_x_*(*x*, *y*) and *G_y_*(*x*, *y*) represent *x*, *y* gradient values, respectively. Then, the edgebox operation is performed on the image. The result of the edgebox is utilized as the weight to rescale the gradient value, and the rest of the patches would be set to 0, thereby increasing the diversity of distributions (i.e., *N − K*). Similarly, the gradient image is divided into image patches and expanded as *k* × *k* × *d* column vectors. Equation 11 is utilized as the distance metric function, and $e^{\varphi <s_{i}^{c}\cdot t_{i}^{c}>}$ is used as a multiplier to reduce the impact of image patches of similar color but different texture.

$$d_{\mathrm{ij}}=\frac{{\left\|s_{i}^{c}-t_{i}^{c}\right\|}_{2}^{2}+\lambda {\left\|s_{i}^{p}-t_{i}^{p}\right\|}_{2}^{2}}{e^{\varphi <s_{i}^{c}\cdot t_{i}^{c}>}},$$ (11)

where $s_{i}^{c}$ is the feature vector of the candidate area, $t_{i}^{c}$ is the feature vector of the template, the corner mark *c* represents the channel index, *d_ij_* is the distance between $s_{i}^{c}$ and $t_{i}^{c}$, < · > denotes the inner product operator between feature vectors, and *φ* is a constant.

It can be seen that the point sets that contain more target distributions would have a larger BBS response. With the proposed caching scheme, the search window can start from any position and move along any adjacent directions. Therefore, the BGD method is introduced to search the maximum response value.

### Scale estimation method

**Fig. 3.**
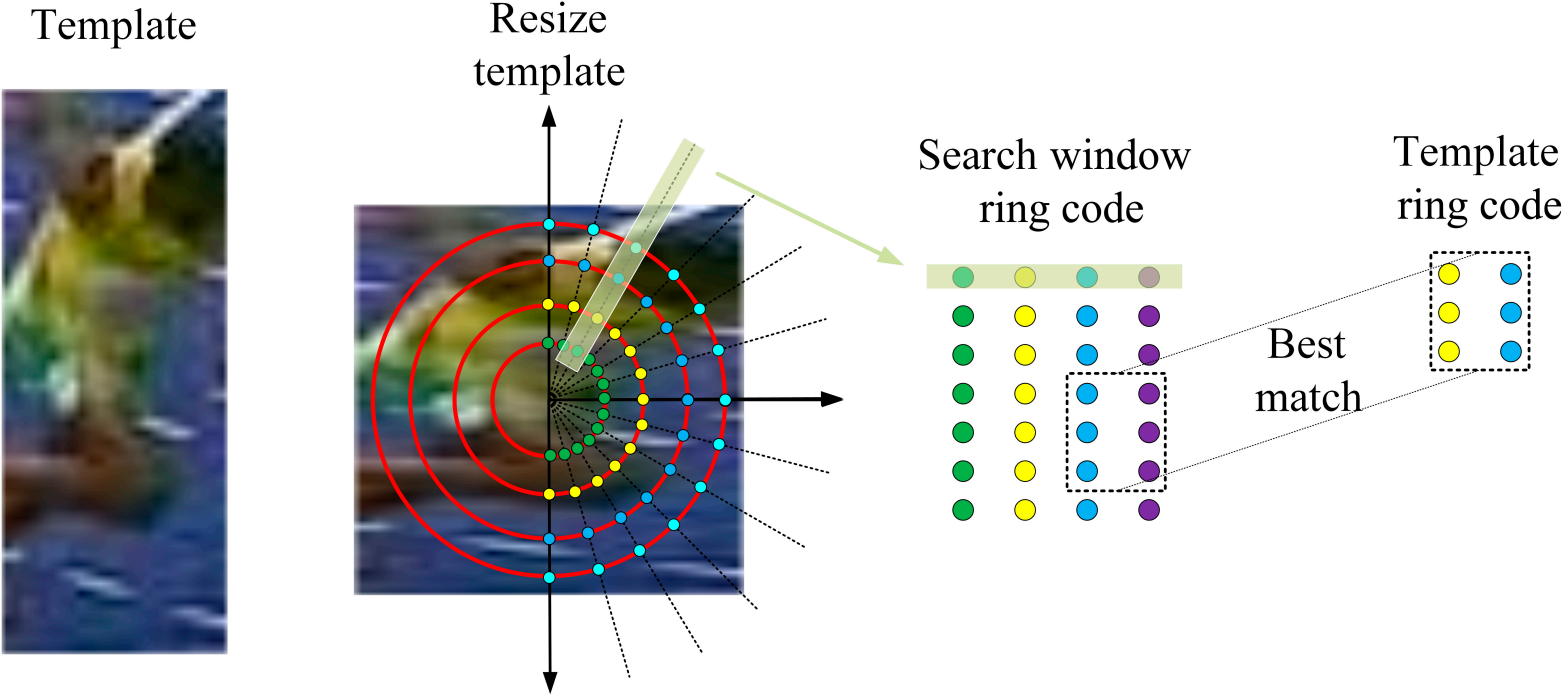
An overview of the scale estimate method.

It is notable that the original BBS can only detect the spatial position of the target and cannot handle scale variation. To address this issue, image patches along the radial tangent direction will be sampled and arranged as a new image (similar to polar coordinate transformation). Pixels within a certain angle range will be sampled with a purpose to simplify computation. *S* point sets along the radial direction will be collected, and the proposed method is utilized to match the displacement along the radial direction, so as to obtain the scale and rotation changes of the target. The overview of the scale estimation method is demonstrated in Fig. 3. 


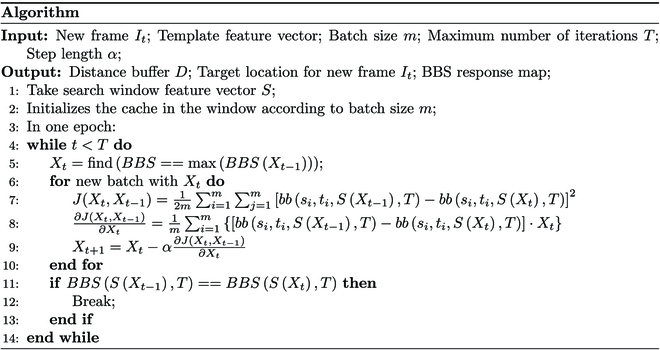


The size of the template and the candidate area is given by a rectangular size. It is not conducive to match template in scale space if the aspect ratio of size is variable. Therefore, first, the candidate area and template are uniformly resized to a certain square size *L* × *L*. The template sampling process will be used as an example. First, a set of concentric circles is defined, and the radius of each circle is arranged in an arithmetic sequence. The parameter *Δr*, which represents the growth value of the concentric circle radius, is introduced so that the number of concentric circles *C* is computed as follows,

$$C=\frac{L-\Delta r}{\Delta r}.$$ (12)

Points on an arc of each group of concentric circles are sampled evenly (mainly to obtain a small point set). If the number of points is insufficient, then it will be obtained by linear interpolation. Before sampling, all images are adjusted to a uniform size; hence, the points to be sampled are also in a certain position that can be precomputed. Thereby, positions to be sampled are precomputed as a lookup table corresponding to Δ*r*. After sampling, all points are rearranged according to sampling order. The displacement of the template in the horizontal direction corresponds to scaling, and the displacement in the vertical direction represents rotation. Assume that the displacement of the template is [Δ*x*, Δ*y*], and only Δ*x* is adopted (the rotation positioning error is unacceptable in validation experiments, so only the scaling results are retained), the scale factor *ℏ* is obtained by:

$$\hbar =\frac{\sqrt{w_{t-1}^{2}+h_{t-1}^{2}}+2\Delta r\Delta x}{\sqrt{w_{t-1}^{2}+h_{t-1}^{2}}}.$$ (13)

The assumption that the target scale does not change drastically is introduced. Therefore, if the *ℏ* changes drastically, then it seems to be an outlier and will be excluded,

$$\begin{array}{c} \text{if}\;\hbar \leq \epsilon ,\text{then}\;\hbar =\hbar \\ \text{else},\hbar =1\\ s_{t}^{l}=\hbar s_{t-1} \end{array}$$ (14)

where $s_{t}^{l}=\left[w_{t-1},h_{t-1}\right]$ denotes the temp size of the bounding box at *t* frame. To improve the robustness of the scale estimation, the target scale *s_t_* is updated by linear interpolation,

$$s_{t}=\left(1-\mu \right)s_{t}^{l}+\mu s_{t-1}.$$ (15)

where *μ* is the linear interpolation constant.

### Template update

A variety of different template update strategies have been tested with the proposed method, and all of them would degrade the tracking performance. Instead, not updating the template and retaining the original information of the target would have a better performance.

## Results and Discussion

To evaluate the performance of the proposed tracker, a caching scheme comparison experiment, an ablation experiment, and a comparison experiment are conducted. All experiments are implemented in MATLAB with an Intel 2.50 GHz CPU, 16 GB RAM, and a Windows 10 x64 operating system. The performance of all trackers are evaluated in 2 aspects [27]. One widely adopted evaluation metric is precision. Precision is the center location error, which is defined as the average Euclidean distance between the center of the tracking bounding box and the manually labeled ground truths. Another evaluation metric is the bounding box overlap. Overlap is the tracked bounding box *r_t_* and the ground truth bounding box *r_a_*; the overlap score is defined as $S=\frac{\left|r_{t}\cap r_{a}\right|}{\left|r_{t}\cup r_{a}\right|}$, where ∩ and ∪ represent the intersection and union of 2 regions, respectively, and |·| denotes the number of pixels in the region. For success plots, area under curve scores are used to summarize and rank the trackers in success plot, while for precision plots, the results at an error threshold of 20 is used for ranking.

### Caching scheme comparison

**Fig. 4.**
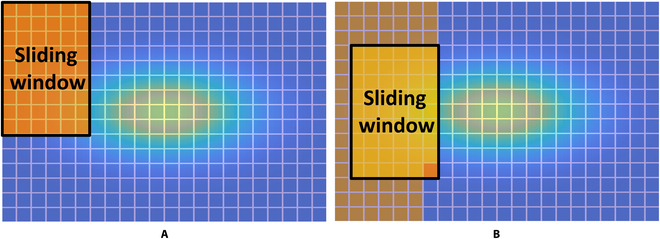
Schematics of the run time experiment setup. (A and B) The computation time of all image patches in the original sliding window (first step) and the computation time of a single image patch when the sliding window moves (per block), respectively.

**Fig. 5.**
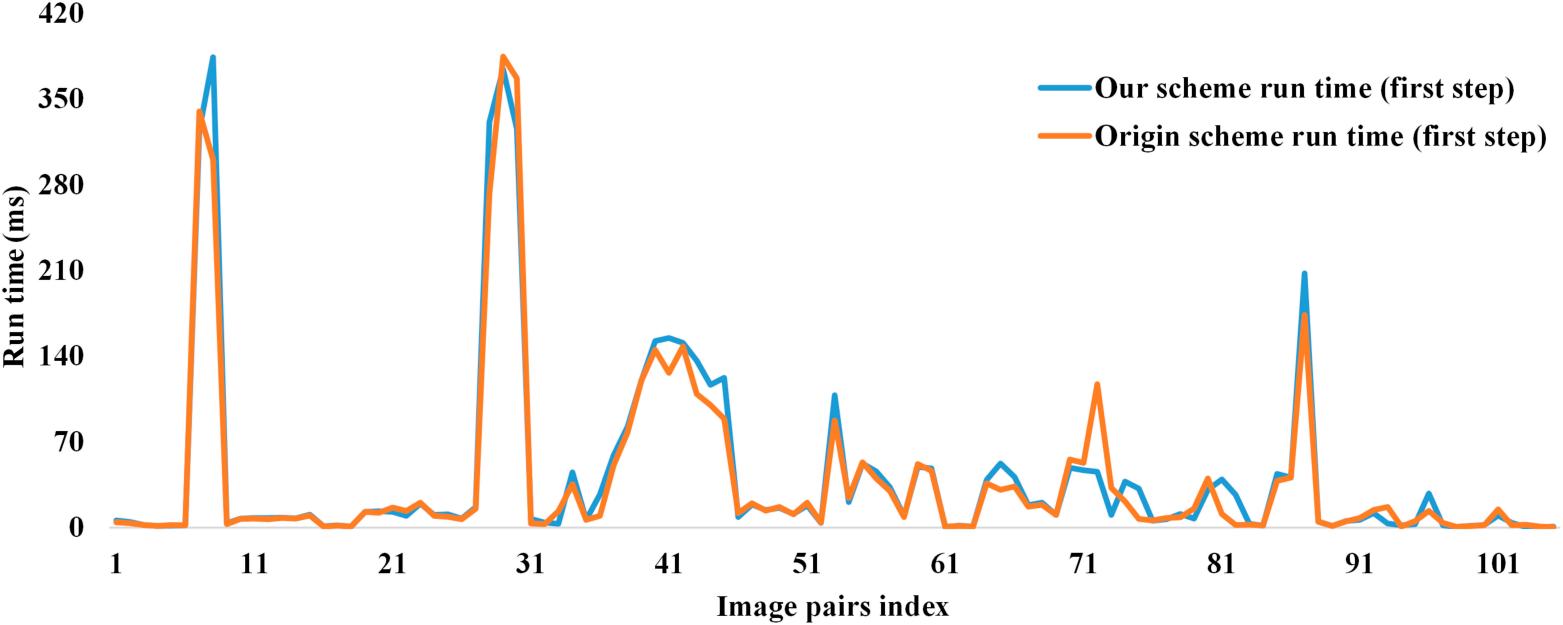
The run time of the first sliding step (a complete initialization process). The orange line is the original caching scheme, and the blue line is our caching scheme.

**Fig. 6.**
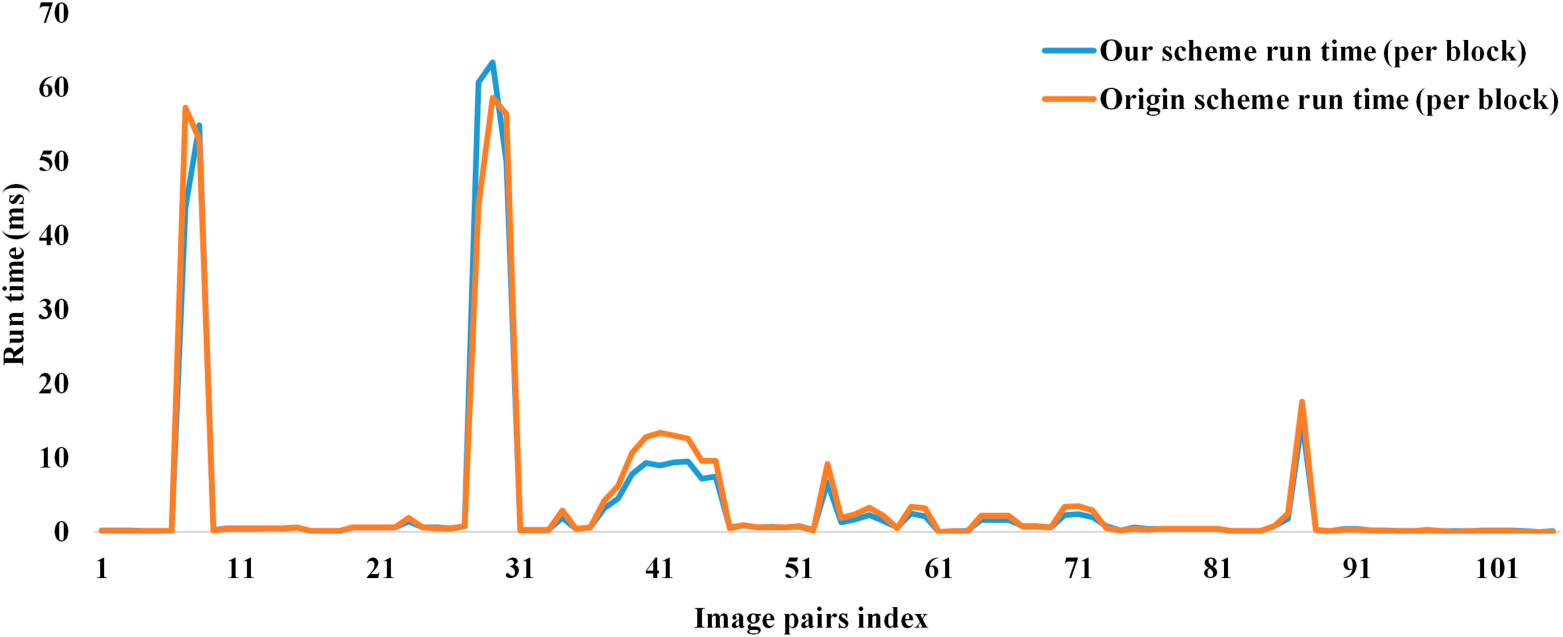
The run time of a normal sliding step. The orange line is the original caching scheme, and the blue line is our caching scheme.

**Fig. 7.**
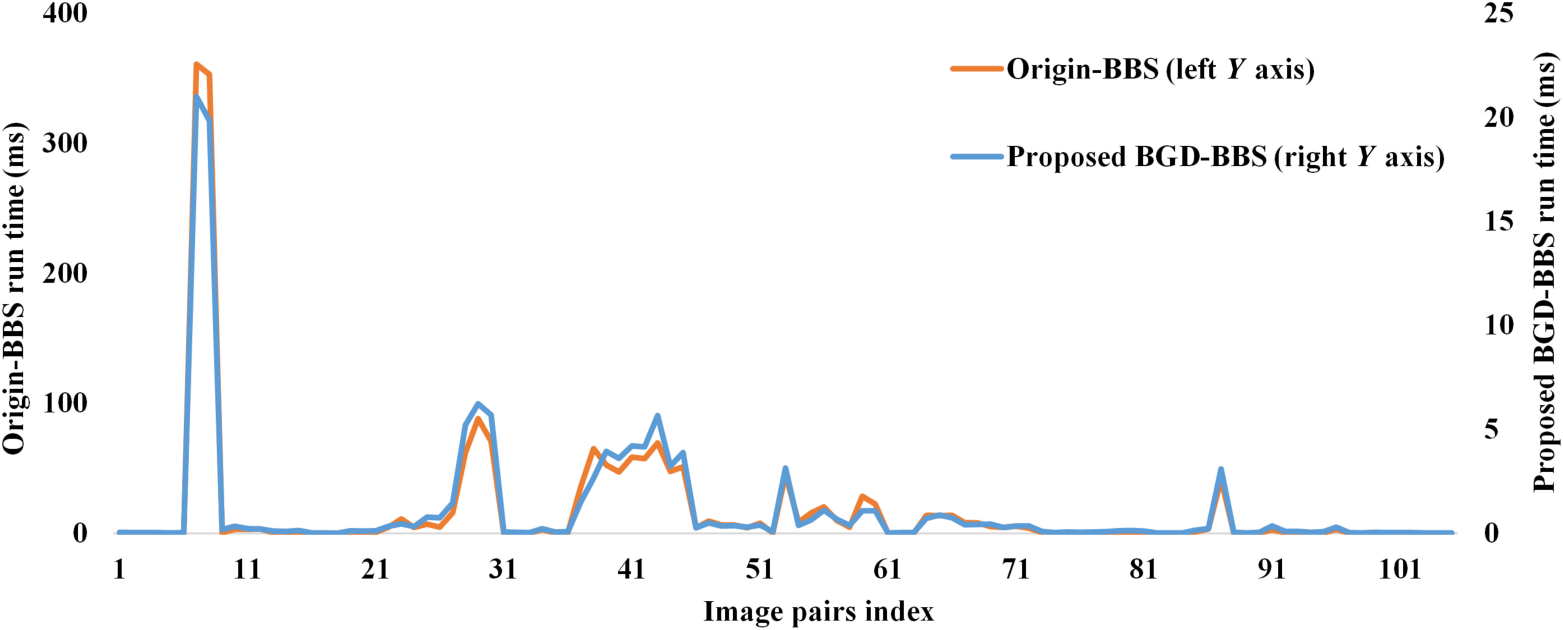
The run time of a complete BBS computation process. The orange line is the original method, and the blue line is the proposed BGD-BBS with a new caching scheme.

To evaluate the proposed caching scheme, extensive experiments on 105 template–image pairs that are adopted in the original work are conducted. All image pairs are selected from the OTB-50 dataset [27]. Time consumption is measured in 3 parts: (a) the computation time of all image patches in the original sliding window (first step), (b) the computation time of a single image patch when the sliding window moves (per block), and (c) the complete computation time. Figure 4 shows the schematic of the sliding run time experiment setup. The complete computation time experiment is set up according to [11]. The experiment results are shown in Figs. 5, 6, and 7, respectively.

As can be seen in Fig. 5, in the sliding window initialization step, the run time of the original caching scheme is in the same order of magnitude as the proposed caching scheme, and it is slightly faster than the proposed caching scheme. Our scheme utilizes a hash map to cache all the image patches. In the initialization step, every patch needs to be cached, so the computation time is slightly higher than the original caching scheme.

In the computation process of a single image patch, the computation time of the proposed cache scheme is faster than the original scheme in Fig. 6, which is also caused by the mapping of the hash map. For a single image patch, it only needs to query the cache result once to get the corresponding distance matrix. At the same time, the proposed caching scheme no longer depends on the moving direction of the sliding window, so it can start from any position of the search window along the 8-neighborhood direction, which is more suitable for visual tracking problems.

Figure 7 demonstrates the run time of the complete BBS computation process. It can be seen from the figure that the BGD-BBS algorithm proposed in this paper runs faster than the original algorithm, and in small-size (30 × 30) template tracking scenarios, our method can achieve close to 30 frames/s. As the template size increases, the run time increases rapidly. In the larger-size (70 × 70) template tracking scenarios, the proposed method can only achieve 3 frames/s. Therefore, in the following video sequence tracking experiments, the template and candidate image will be resized so that the inner product of its size is within 1,000, so as to ensure the real-time performance of the algorithm, although this will slightly degrade the performance of the algorithm.

### Ablation study

**Fig. 8.**
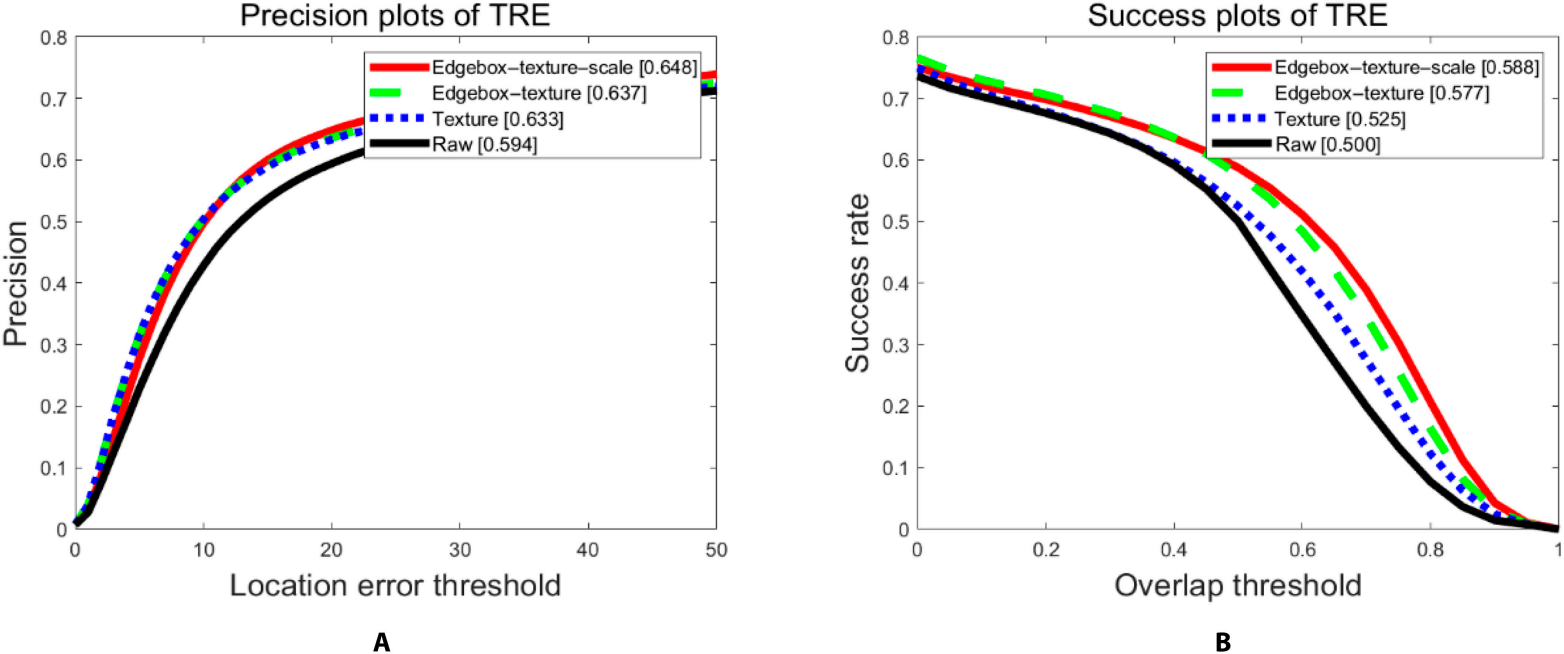
Precision plot and success plot for all 50 sequences. The tracker that enables all components outperforms other component groups. Best viewed in color. (A) Precision plot. (B) Success plot. TRE, temporal robustness evaluation.

The proposed method is divided into 3 modules, namely, the patch-based texture feature module (texture), the distribution dimensionality reduction module (edgebox-texture), and the scale estimation module (scale). The ablation experiment is performed on the OTB-50 dataset with different components enabled. The raw (none) method is the best buddies tracker that only enables the BGD method and the proposed caching scheme. The texture (T) adds a patch-based texture feature to the raw method. Edgebox-texture (ET) adds feature distribution and dimensionality reduction operations on the basis of texture, and edgebox-texture-scale (ETS) denotes the RSBBT, which enables all components. Comparative experiments are conducted on the above 4 different methods to verify the impact of different modules on tracking performance. All sequences are classified to 11 challenging tracking scenarios, which are illumination variation (IV), scale variation (SV), occlusion (OCC), deformation (DEF), motion blur (MB), fast motion (FM), in-plane rotation (IPR), out-of-plane rotation (OPR), out of view (OV), background clutters (BC), and low resolution (LR). The experiment results are shown in Fig. 8 and Tables 1 and 2 below.

**Table 1.** Summary of experimental location error results under the 11 challenging conditions. The reported quantities are averaged over all videos.

Trackers	IV	OPR	SV	OCC	DEF	MB	FM	IPR	OV	BC	LR
ETS	0.503	**0.620**	**0.540**	**0.652**	**0.703**	0.537	**0.532**	**0.598**	**0.513**	**0.577**	0.452
ET	**0.505**	0.603	0.532	0.640	0.692	**0.548**	0.519	0.589	0.486	0.562	**0.466**
T	0.498	0.601	0.525	0.643	0.689	0.537	0.513	0.587	0.503	0.554	0.451
None	0.448	0.581	0.492	0.578	0.684	0.515	0.525	0.552	0.468	0.549	0.444

**Table 2.** Summary of experimental overlap results under the 11 challenging conditions. The reported quantities are averaged over all videos.

Trackers	IV	OPR	SV	OCC	DEF	MB	FM	IPR	OV	BC	LR
ETS	**0.470**	**0.556**	**0.466**	**0.592**	**0.627**	**0.518**	**0.497**	**0.544**	0.475	0.521	**0.439**
ET	0.455	0.549	0.438	0.577	0.617	0.497	0.483	0.522	**0.501**	**0.532**	0.414
T	0.405	0.503	0.390	0.507	0.592	0.468	0.467	0.473	0.463	0.508	0.417
None	0.406	0.464	0.397	0.510	0.527	0.468	0.436	0.454	0.433	0.441	0.411

It can be seen from the above experiment results that each module can improve the performance of the tracker to a certain extent. The tracker that enables all modules achieves the best performance in both location error and overlap experiments. In 11 challenging tracking scenarios, the tracker with all modules enabled achieves the first or the second best performance. It can be seen that only the texture features module cannot improve the performance of the tracker significantly based on the experiment results of the texture module and the raw method. Especially in illumination variation, scale variation, occlusion, and motion blur, the performance drops instead. The texture features are introduced in the texture module, and the clutter background texture is also introduced. The special distribution of the sample is not filtered, and it will cause an increase in the diversity of the distribution of the sample, thereby leading to a mismatch. The performance of the algorithm has been significantly improved (more than 10%) in most tracking scenarios after being added a simple filtering strategy (edgebox-texture). Finally, the scale estimation module is enabled. In most scenarios, the performance of the algorithm has been improved, especially in scale variation scenarios. It can be seen that the proposed method performs best in deformation and occlusion scenarios. The BBS is a similarity measurement method that tends to chi-square similarity, and it is adaptive to deformation and partial occlusion. Similarly, it does not achieve an excellent performance in low-resolution and illumination variation scenarios because of the RGB color feature. The appearance model based on color is difficult to convey the information of the target in the low-resolution image, and the illumination change affects the color-based appearance features greatly.

### Evaluation on sequences

OTB-50 is used for comparison among the proposed RSBBT and other recently published trackers. The performance of our method is compared to other recently published tracker. Specifically, the trackers are as follows: kernelized correlation filter (KCF) [20], color-based probabilistic framework (CPF) [28], circulant structure with kernels (CSK) [29], adaptive structural local sparse appearance model (ASLA) [30], tracking-learning-detection (TLD) [31], online multiple instance learning (MIL) [32], and compressive tracking (CT) [33].

**Fig. 9.**
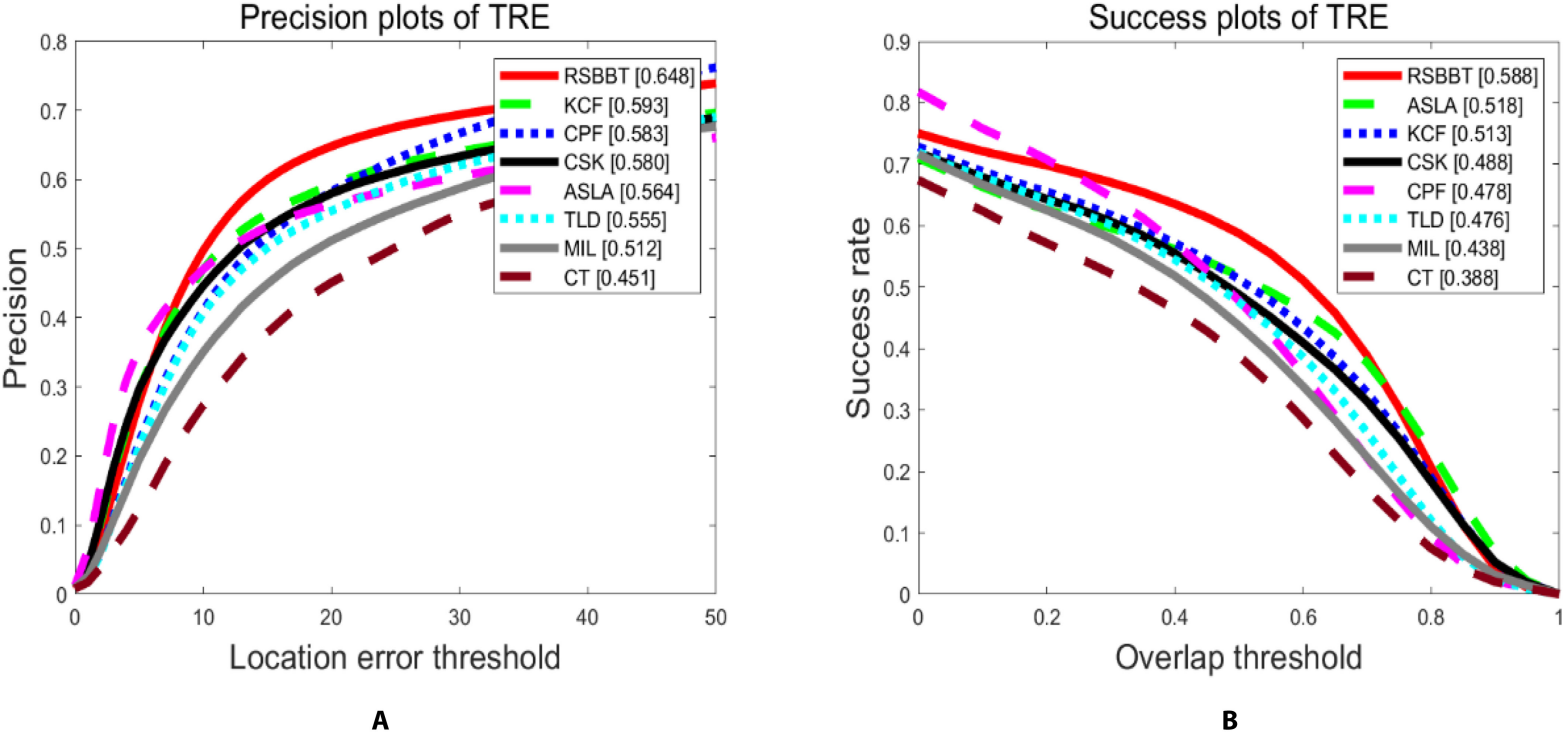
Precision plot and success plot for all 50 sequences. The proposed tracker outperforms other trackers. Best viewed in color. (A) Precision plot. (B) Success plot.

All experiments are conducted with fixed parameters. For KCF, the raw pixel feature is utilized. For RSBBT, *k* used in an image patch size is set to 3, *ϕ* used in Eq. 13 is set to 2, *Δr* used in Eq. 12 is set to 2, the scale update threshold *ϵ* is set to 1.1, and the scale update factor *μ* is set to 0.15. Other parameters not specified were set as those in the original work. The experiment results are shown in Fig. 9 and Tables 3 and 4 below.

**Table 3.** Summary of experimental location precision results under the 11 challenging conditions. The reported quantities are averaged over all videos.

Trackers	IV	OPR	SV	OCC	DEF	MB	FM	IPR	OV	BC	LR
RSBBT (ours)	**0.503**	**0.620**	0.540	**0.652**	**0.703**	**0.537**	**0.532**	**0.598**	**0.513**	**0.577**	0.452
KCF	0.459	0.541	0.542	0.542	0.562	0.459	0433	0.563	0.202	0.540	0.456
CPF	0.447	0.603	0.567	0.603	0.666	0.393	0.454	0.509	0.436	0.485	0.219
CSK	0.491	0.539	0.544	0.563	0.549	0.391	0.358	0.518	0.225	0.540	**0.482**
ASLA	0.502	0.542	**0.599**	0.542	0.554	0.335	0.281	0.500	0.204	0.543	0.325
TLD	0.448	0.539	0.581	0.558	0.568	0.461	0.466	0.474	0.382	0.453	0.376
MIL	0.385	0.483	0.471	0.482	0.577	0.375	0.359	0.464	0.287	0.491	0.326
CT	0.375	0.432	0.472	0.462	0.529	0.294	0.299	0.388	0.238	0.400	0.234

**Table 4.** Summary of experimental overlap results under the 11 challenging conditions. The reported quantities are averaged over all videos.

Trackers	IV	OPR	SV	OCC	DEF	MB	FM	IPR	OV	BC	LR
RSBBT (ours)	**0.470**	**0.556**	0.466	**0.592**	**0.627**	**0.518**	**0.497**	**0.544**	**0.475**	**0.521**	**0.439**
KCF	0.391	0.462	0.423	0.446	0.484	0.421	0.399	0.492	0.210	0.488	0.414
CPF	0.341	0.508	0.435	0.511	0.528	0.318	0.391	0.415	0.388	0.385	0.163
CSK	0.403	0.444	0.413	0.453	0.473	0.359	0.320	0.436	0.221	0.473	0.405
ASLA	0.456	0.481	**0.543**	0.499	0.513	0.298	0.251	0.441	0.205	0.483	0.304
TLD	0.374	0.460	0.469	0.479	0.488	0.427	0.413	0.387	0.360	0.392	0.324
MIL	0.334	0.398	0.363	0.417	0.509	0.320	0.317	0.366	0.298	0.429	0.299
CT	0.331	0.360	0.364	0.394	0.482	0.246	0.251	0.304	0.239	0.359	0.197

The results of various tracking scenarios show that the proposed method can achieve the best performance in most scenarios, and it excels in occlusion, deformation, motion blur, fast motion, and out of view scenarios. The BBS compares the image patch with the entire template and counts the BBP, which makes the tracker robust to lack of global information. Moreover, the proposed method employs the BGD method, which can search for the target in a large range efficiently. However, in illumination change, low-resolution, and scale variation scenarios, the proposed method does not achieve the best performance. The proposed method utilizes color-based appearance model, which is sensitive to illumination change and conveys less information in tracking scenarios such as low resolution and scale variation. Therefore, other types of features that convey more information will improve the performance of our method in these challenging scenarios.

**Fig. 10.**
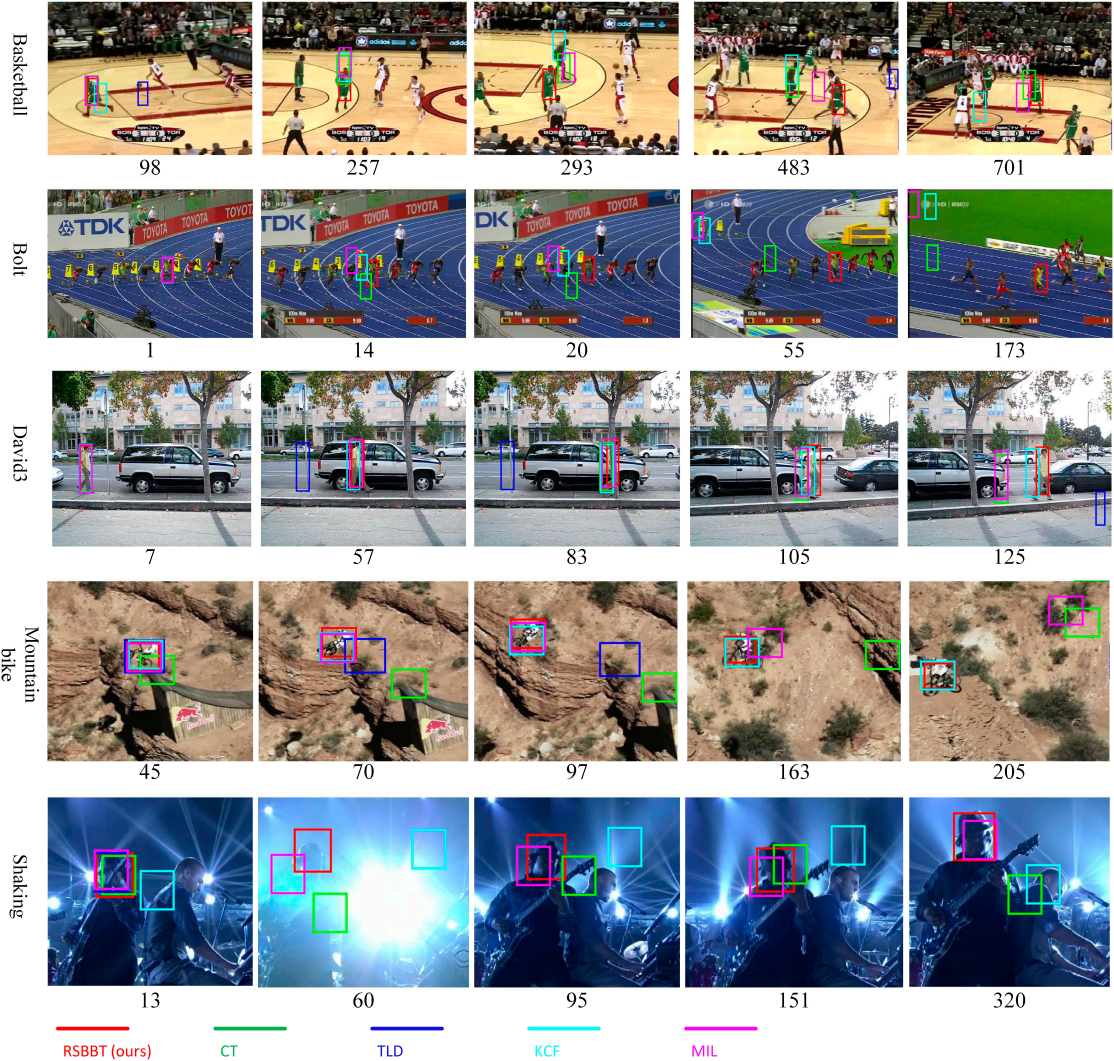
Tracking sequences for evaluation. Illustration of the qualitative tracking results on challenging sequences (top to bottom: basketball, bolt, David3, mountain bike, and shaking). The color bounding boxes are the corresponding results of the indicated legends.

The proposed method is compared with other state-of-the-art trackers, such as KCF [20], TLD [31], and CT [33] on several challenging sequences from the OTB-50 dataset [27]. Figure 10 shows some representative examples to demonstrate the advantages of the proposed method. The proposed method can handle nonrigid deformation, motion blur, occlusion, and scale variation of the target better than other trackers. For example, in the basketball sequence, RSBBT can track the target accurately despite its deformation and occlusion by other players. In contrast, other trackers lose the target or drift to other objects. In the bolt sequence, RSBBT can handle the motion blur and deformation of the target, while other trackers gradually drift and eventually fail. In the David3 sequence, RSBBT and KCF can track the target under deformation and occlusion, but KCF suffers from boundary effects, which cause the bounding box to shift. In the mountain bike sequence, RSBBT and KCF can handle the rapid rigid deformation of the target, while other trackers cannot adapt to the scale change. One of the reasons why RSBBT outperforms other trackers is that it does not update the template of the target, which avoids introducing errors or noise into the model. Instead, it relies on the robustness of BBS to nonrigid deformation, which can find the best matching between the template and the candidate image based on point sets. Another reason is that RSBBT introduces patch-based texture features to cope with cluttered backgrounds, which can cause local multiple response peaks and impair the accuracy of BBS. With patch-based texture features, the peak of the response value is steeper and more distinctive. Moreover, RSBBT uses an improved caching scheme and a BGD algorithm to improve the computation efficiency of BBS, which makes it more suitable for real-time visual tracking applications.

## Conclusion

In this paper, a real-time scale-adaptive visual tracking method based on BBS is presented. The original work is improved by modifying the caching scheme of the original algorithm so that it does not depend on the sliding direction of the sliding window and can search for the maximum response position along any direction of the 8-neighborhood direction. The BGD method is employed to reduce the computational complexity. The effect of the cluttered background on the BBS response value is theoretically analyzed, and a simple strategy is proposed to achieve the dimensionality reduction of the distribution. The analysis is validated through ablation experiments. The search window and template are resampled, and the proposed method is applied to search in scale space. Experimental results show that the proposed method can handle a variety of challenging tracking scenarios and is more robust to some scenarios such as deformation and partial occlusion.

## Data Availability

The data that support the findings of this study are available from the corresponding author upon reasonable request.
